# Liver-Protective Effects of Hydroalcoholic Extract of *Allium Hirtifolium* Boiss. in Rats with Alloxan-Induced Diabetes Mellitus

**Published:** 2010

**Authors:** Somayeh Kazemi, Sedigheh Asgary, Jamal Moshtaghian, Mahmoud Rafieian, Azadeh Adelnia, Fatemeh Shamsi

**Affiliations:** 1 Department of Biology, School of Sciences, The University of Isfahan and Isfahan Cardiovascular Research Center, Isfahan University of Medical Sciences, Isfahan, Iran; 2 Isfahan Cardiovascular Research Center, Applied Physiology Research Center, Isfahan University of Medical Sciences, Isfahan, Iran; 3 Department of Biology, School of Sciences, The University of Isfahan, Isfahan, Iran; 4 Medicinal Plants Research Center, Shahrekord University of Medical Sciences, Shahrekord, Iran; 5 Department of Biology, School of Science, Isfahan Payam-e-Noor University, Isfahan, Iran

**Keywords:** Diabetes, Allium hirtifolium, Shallot, Alloxan monohydrate, Liver, Rat

## Abstract

**BACKGROUND:**

Diabetes mellitus is one of the most common endocrine disorders accompanied with many metabolic syndromes. Use of herbal medicines has always been an option to treat a great number of diseases such as diabetes and its complications. In this study the liver-protective effects of hydroalcoholic extract of *Allium hirtifolium* on liver enzymes level in rats with alloxan-induced diabetes mellitus was investigated.

**METHODS:**

Thirty five male rats were randomly divided into five groups of seven; group 1: nondiabetic control, group 2: diabetic control, group 3: diabetic treated with shallot extract (0.1 g/kg), group 4: diabetic rats treated with shallot extract (1 g/kg), and group 5: diabetic treated with glibenclamide (0.6 mg/kg). Using intraperitoneal (IP) injection of alloxan monohydrate, diabetes mellitus was induced in rats. Diabetic rats were treated with intraperitoneal injection for 4 weeks. At the end of the experimental period fasting blood samples were collected.

**RESULTS:**

Statistical analysis of the data indicated that hydroalcoholic extract of shallot can significantly decrease serum contents of liver enzymes (ALP, AST, and ALT) in treated groups. In most cases, the effectiveness of the extract on reduction of these enzymes is more than glibenclamide.

**CONCLUSION:**

Antioxidant compounds in the extract may recover liver damages caused by free radicals in diabetic rats.

## Introduction

Diabetes mellitus is a common metabolic disease which is a result of either a deficiency in insulin secretion, or insulin resistance in body cells. Diabetes-induced hyperglycemia and hyperlipidemia can cause many structural and metabolic disorders in different body organs including the liver.^1^, ^2^ Considering the great developments in human knowledge about diabetes mellitus diversity and knowing that it is one of the major health, social and economical problems in the world, a need for finding effective compounds, with fewer side effects, to treat diabetes and its complications has been arisen.^3^ Although medicinal herbs and their derivatives have been used as a remedy for diabetes mellitus for a long time, their certain effectiveness has not yet been proven by any valid research.^4^ Shallot, scientifically called *Allium hirtifolium* Boiss., belongs to *Allium* genus and liliaceae family. Garlic, onion, shallot and leek are the most important species in this genus and have been long used as spices and also medicine.^5^ Saponins, sapogenins, sulphuric compounds (thiosulfinates) and flavonoids, including quercetin and kaempferol, are found in different species of *Allium* genus. Research has shown that both the bulb and the flower of shallot contain a high density of glycosidic flavonols.^6^, ^7^ Disulphide and trisulphide compounds are amongst the most important compounds existing in *Allium* genus species.^8^ There have been reports about shallot having pharmacological effects, e.g. antioxidant.^9^ immune system regulating,^10^ anticancer^11^ and anti-trichomonas^12^ effects; however, no official report exists about the usefulness of shallot in improving liver function. Therefore, the present study mainly aimed to examine the liver-protective effects of hydroalcoholic extract of *Allium hirtifolium* Boiss. on liver enzymes in rats with alloxan-induced diabetes mellitus, and also to compare its effectiveness with glibenclamide medicine.

## Materials and Methods

Bulbs of shallot were purchased at Medicinal Plants Research Division, Isfahan's Department of Natural Resources, in May 2008. The genus and species of the bulbs were confirmed by the botanists at Department of Biology, Isfahan University. Herbarium specimens of shallot, numbered 1583, are kept at medicinal plants herbarium in Department of Pharmacy, Isfahan University of Medicine.

### Hydroalcoholic extract preparation method

First, the plant was changed into a powder which was then poured into a 1 liter Erlenmeyer and ethanol (90%) was added until it covered the powder. After 48 hours the solution was filtered using a filter paper and a Buchner funnel. In the second phase, ethanol (50%) was added to the remaining pulp and the previous stages were repeated. Then the filtered solutions obtained from the first and second stages were mixed and concentrated to 1/3 of original volume by means of a vacuum distillation unit at the speed of 70 rpm and 60°C and decanted for several times by using chloroform. The solution obtained from the last stage was dried in an autoclave under sterile conditions at 50°C. After a few days dry powder of the extract was ready and kept at 4°C.^10^ Using this method 51.9 g extract was obtained from 100 g powder.

### Experimental animals

Thirty five white, male Wistar rats, weighing 190–220 g, were used in this study. The animals were purchased from Ahvaz University of Medicine (Jondi Shapour) and were kept in the animal lair at Department of Biology, Isfahan University, under appropriate temperature, humidity and light conditions. All the experiments were postponed until after 2 weeks in order for the rats to become accustomed to the new environment. The rats could freely access water and the special food prepared for them. The present study has been confirmed by ethics committee of Isfahan Cardiovascular Research Center.

### Induction of diabetes

The experimental model of diabetes mellitus type 1 (insulin dependent diabetes) was induced by a one-time intraperitoneal (IP) injection of solution of alloxan monohydrate (Sigma, Germany) in normal saline at a dose of 120 mg/kg of body weight.^13^, ^14^ After 72 hours of alloxan injection, fasting blood sugar was measured in rats using a glucometer to determine whether they were diabetic or not.^15^ The criterion for diabetes was having fasting glucose level of higher than 130 mg/dl.^16^

### Categorization

In this study 35 rats were randomly divided into 5 groups of 7:

- Group 1: Nondiabetic control group who received normal saline intraperitoneally for 4 weeks (the amount of injected saline was equal to the amount of injected extract). This was only done to equalize the injection shock in all groups.
- Group 2: Diabetic control group who were treated with normal saline for 4 weeks.
- Group 3: Diabetic group who had intraperitoneal injections of the extract solved in normal saline, at a dose of 0.1 g/kg of body weight for 4 weeks.
- Group 4: Diabetic group who had intraperitoneal injections of the extract solved in normal saline, at a dose of 1 g/kg of body weight for 4 weeks.
- Group 5: Diabetic group who had intraperitoneal injections of glibenclamide medicine (Hakim Pharmacy, Iran) solved in normal saline, at a dose of 0.6 mg/kg of body weight for 4 weeks.

### Blood sampling and biochemical tests

After the treatment period, blood sampling was performed on the rats and alanine aminotransferase (ALT), aspartate aminotransferase (AST), and alkaline phosphatase (ALP) enzymes levels were measured using a Pars Azmoon kit (Iran) and a Hitachi 902 Automatic Analyzer. Sixteen hours before blood sampling food was taken out of rats' reach.^14^, ^15^

### Statistical analysis of data

The results were statistically analyzed as mean ± SEM. Kruskal Wallis test was used to compare the results for each parameter. The significance level of P < 0.05 was chosen for all the analyses.

## Results

The present study has measured serum liver enzymes in rats at the end of the experimental period and the following results were obtained (Table 1):

**Table 1:** Effect of hydroalcholic extract of *A hirtifolium* on liver enzymes levels in diabetic rats

Group	ALP (u/l)	ALT (u/l)	AST (u/l)
Normal control	349.57 ± 23.63*†	49.71 ± 2.05*	142.42 ± 12.26*
Diabetic control	1244.42 ± 92.24†	66.42 ± 6.79†	174.85 ± 5.94†
Diabetic + shallot extract (0.1 g/kgBW)	823 ± 278.68*†‡	49.8 ± 7.49*	143 ± 12.6*‡
Diabetic + shallot extract (1 g/kgBW)	490.71 ± 91.5*†	41.85 ± 2.36*†	103 ± 8.87*†
Diabetic + glibenclamide (0.6 mg/kgBW)	1025.5 ± 189.69*	58.83 ± 7.31	156.74 ± 10.96*

Values are presented as mean ± SEM (n = 7)

* Significantly different from diabetic control (P < 0.05)

† Significantly different from diabetic + glibenclamide (P < 0.05)

‡ Significantly difference between high dose and low dose of extract (P < 0.05)

### A. The effects of hydroalcoholic extract of Allium hirtifolium on aspartate aminotransferase (AST) levels

The results of statistical analysis show a significant difference in the mean value of AST levels in shallot extract-treated and diabetic control groups (P < 0.05).

This difference indicates the effectiveness of shallot in reducing blood AST levels. As it is seen in table 1, the effectiveness of shallot extract significantly changes based on its dosage (P < 0.05) and the high dose of the extract (1 g/kg) has even been more effective than glibenclamide in decreasing AST levels (P < 0.05).

### B. The effects of hydroalcoholic extract of Allium hirtifolium on alanine aminotransferase (ALT)

The significant difference between shallot extract-treated and diabetic control groups confirms that shallot could dramatically decrease ALT levels in rats. As shown in table 1, both the high and low dosages of shallot extract were equally effective in reducing ALT levels and no significant difference has been observed among them (P < 0.05). They were also better than glibenclamide medicine and significant difference in mean values has been noticed between high dose of the extract (1 g/kg) and glibenclamide-treated groups (P < 0.05).

### C. The effects of hydroalcoholic extract of Allium hirtifolium on alkaline phosphatase (ALP)

As seen in table 1, shallot (both high and low doses) could significantly reduce ALP as compared with diabetic control group (P < 0.05). Shallot extract, either in low (0.1 g/kg) or high (1 g/kg) doses, has been more effective than glibenclamide medicine in reducing ALP and a significant difference has been observed between shallot extract-treated and glibenclamide-treated groups (P < 0.05). The significant difference between mean of the high and low dosages of shallot extract indicates that in diabetic rats, the high dose has been more useful in decreasing ALP levels to a value close to those of healthy control rats (P < 0.05).

## Discussion

Reports about diabetes show that the oxidative stress caused by free radicals significantly decreases antioxidant activity, and as seril theory suggests, hyperglycemia, which damages the liver tissue, is a vital factor for oxidative stress.^17^ The increase in serum liver enzymes (ALT, AST, and ALP) levels, which is probably a result of the enzymes leaking from liver cells cytosol into bloodstream, is an indicator of liver damage.^18^ Larcan et al reported that necrosis of liver tissue is observed in diabetic subjects.^19^ A number of researches have reported the effects of plants in the *Allium* genus, including onion and garlic, on diabetes induced-liver damage, but no reports exist about the effectiveness of shallot. A study conducted by El Demerdash et al suggested that liver enzymes levels change considerably even in alloxan-induced diabetes, and that using onion or garlic extract can be helpful in reducing these enzymes.^20^ Eidi et al also suggested that in diabetic rats, consuming garlic extract decreases liver enzymes levels.^21^ The present study revealed that treating rats with hydroalcoholic extract of shallot could protect liver cells against oxidant effects of alloxan, and it could consequently cause a significant reduction in serum concentration of ALP, ALT, and AST as compared with the diabetic control group. In addition, similar to the results of studies performed by Subash et al and Lukmanul Hakkim et al, glibenclamide injection was also effective on reducing these indices toward normal.^22^, ^23^ Biochemical results confirm the usefulness of shallot extract in decreasing the destructive effects of alloxan on liver tissue and hence on reducing the enzymes' leak into cytosol, which is possibly a result of herbal antioxidant compounds. Polyphenolic compounds and flavonoids can protect the cells against emptying of reduced glutathione by increasing antioxidant enzymes capacity (such as catalase, superoxide dismutase and glutathione peroxidase).^24^ Furthermore, having antioxidant properties, these compounds are able to neutralize free radicals existing in the environment and prevent their destructive effects.^25^ Flavonoids, such as quercetin, inhibit glucose absorption in the intestines and therefore, have hypoglycemic activities.^26^, ^27^ The *Allium* genus contains other effective compounds, including active sulphur compounds, to which shallot's usefulness may be associated. -SH groups of sulphur compounds oxidize the lipid-synthesizing enzymes, and hence reduce or inhibit lipid synthesis. Moreover, these compounds oxidize NADPH to NADP, and since NADPH provides the hydrogen necessary during lipid synthesis stages, they inhibit lipid synthesis.^28^ Sulphur compounds also increase the activity of 7-alpha hydroxylase enzyme, and therefore increase the conversion of cholesterol into bile acids.^29^ So, hydroalcoholic extract of shallot can possibly decrease plasma liver enzymes levels through the reduction of liver cell damage, and also through hypoglycemic and hypolipidemic activities and preventing fatty liver formation.
